# Mitochondria, metabolism and cancer: a growing role in cancer cell differentiation and cancer cell dormancy

**DOI:** 10.1186/2049-3002-2-S1-P64

**Published:** 2014-05-28

**Authors:** Roberto Scatena, Patrizia Bottoni, Bruno Giardina

**Affiliations:** 1 Institute of Biochemistry and Clinical Biochemistry - Catholic University, Rome, Italy

## Background

It is well known that physical or chemical agents can induce cell differentiation, or better pseudo-differentiation, in different human tumor cell lines. As an example, we cite the synthetic ligands of Peroxisome Proliferators Activated Receptors (PPARs), a very heterogeneous class of chemical with numerous biological activities which are mainly reported for their capacity to bind and activate this particular class of nuclear receptors. PPARs are involved in lipid and glucidic metabolism, immune regulation, cancer cell differentiation and, paradoxically, cancer induction. As a matter of fact, some synthetic PPAR-ligands have been already employed in pharmacotherapy and all showed intriguing pharmacotoxicological profiles. A re-evaluation of the biological activities of PPARs synthetic ligands, in particular of the induced mitochondrial dysfunction based on a rotenone-like Complex I partial inhibition and of its consequent metabolic adaptations seems to shed new light on dedifferentiation/differentiation processes in cancer and above all in cancer cell dormancy.

## Material and methods

Adopting different biochemical and proteomic approaches, we investigate the effect of some PPAR-agonists on human hepatocellular carcinoma Hep-G2 cell line. Cancer differentiation was assayed by checking albumin, transferrin and a-fetoprotein synthesis. Cell oxidative metabolism was monitored by high-resolution respirometry.

## Results

Results seem to confirm a significant role of mitochondria in the regulation of differentiation/dedifferentatiation state of cancer cell. Specifically, the energy deprivation related to mitochondria derangement seem to determine a pseudo-differentiation state associated to a condition of cancer dormancy. Moreover these cell seem to assume some biological characteristics of the so-called cancer stem cells.

## Conclusions

Elucidating the relationship between cancer cell oxidative metabolism and cellular differentiation state is fundamental not only to understand the molecular mechanisms linking cancer, mitochondria and metabolism but also to clarify the real pharmacotoxicological profile of the new antimetabolite drugs (i.e., glycolytic enzyme inhibitors) in cancer treatment.

